# Supplementary material for: The sugar-responsive enteroendocrine neuropeptide F regulates lipid metabolism through glucagon-like and insulin-like hormones in Drosophila melanogaster
Source: Nat Commun. 2021 Aug 10;12:4818. doi: 10.1038/s41467-021-25146-w (PMC8355161; doi:10.1038/s41467-021-25146-w)
Supplement: Supplementary file 1 — Supplementary Information File (Supplementary Figures 1-15; Legends for Supplementary Data 1-6) [file 41467_2021_25146_MOESM1_ESM.pdf]

Supplementary Information for

The sugar-responsive enteroendocrine neuropeptide F regulates lipid metabolism through glucagon-like and insulin-like hormones in *Drosophila melanogaster*

Yuto Yoshinari, Hina Kosakamoto, Takumi Kamiyama, Ryo Hoshino, Rena Matsuoka, Shu Kondo, Hiromu Tanimoto, Akira Nakamura, Fumiaki Obata, and Ryusuke Niwa.

This file contains: Supplementary Figs. 1 – 15

Legends for Supplementary Data 1 – 6

Supplementary Fig. 1

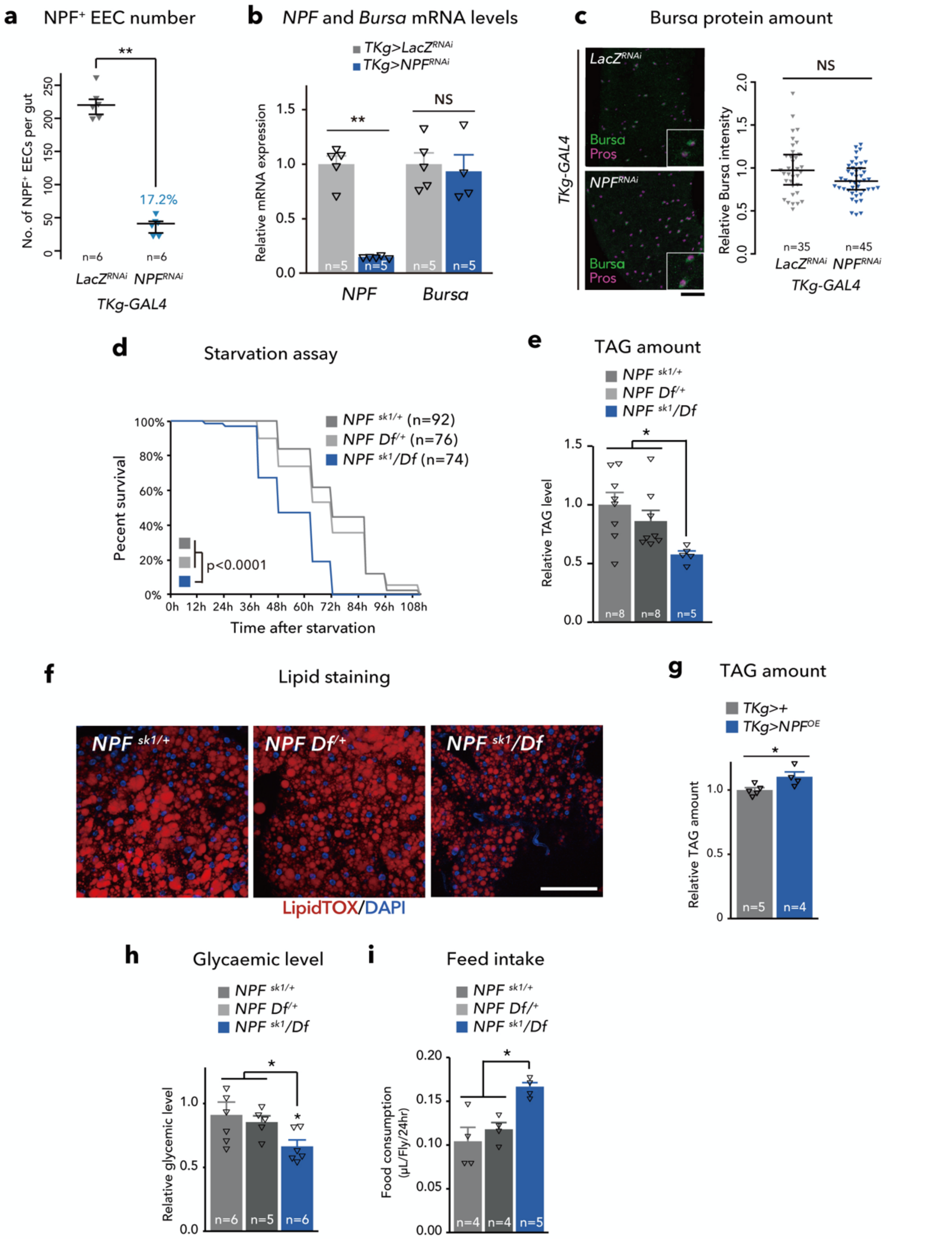

Supplementary Fig. 1. Midgut-specific *NPF* knockdown reduced the mRNA expression of *NPF*, but not *Bursα*.

a, Number of NPF^{+} EECs per midgut. n = 6 samples in each genotype. The number of samples assessed (n) are indicated in the graphs. **b**, Relative change in the mRNA levels of neuropeptide genes in the gut. The number of samples assessed (n) is indicated in the

graphs. **c**, Immunostaining for Bursα (green) and Prospero (magenta) and quantifications of Bursα fluorescent intensity in adult posterior midguts of control (*TKg>LacZ*^{RNAi}) and *NPF* knockdown (*TKg>NPF*^{RNAi}) animals. The number of EECs analysed in each genotype is indicated in the graph. Each point represents Bursα fluorescent intensity in a single EEC. For each genotype, we used more than seven guts. Scale bar, 50 µm. **d**, Survival during starvation in flies of each genotype. The number of animals assessed (n) is indicated in the graphs. **e, g**, Relative TAG levels of each genotype. The number of samples assessed (n) is indicated in the graphs. **f,** LipidTOX (red) and DAPI (blue) staining of dissected fat body tissue of NPF mutant. Scale bar, 50 µm. h, Relative glycaemic level of NPF mutant animals. The number of samples assessed (n) is indicated in the graphs. **i,** Feeding amount measurement with CAFÉ assay. The number of samples assessed (n) is indicated in the graphs. For all bar graphs, mean and SEM with all data points are shown. For all dot blots, the three horizontal lines on each sample indicate lower, median, and upper quartiles. Statistics: Wilcoxon rank sum test (a, c), two-tailed Student's *t*-test (b, g), Log rank test (d), two-tailed Student's *t*-test with Holm's correction (e, h, and i). *$p < 0.05$, **$p < 0.01$; NS, non-significant ($p > 0.05$). *p*-values: **a**, $p < 0.0001$; **b**, (*NPF*) $p < 0.0001$, (*Bursα*) $p = 0.7248$; **c**, $p = 0.053$; **d**, $p < 0.0001$ ($NPF^{sk1/+}$ vs NPF^{sk1}/NPF^{Df}), $p < 0.0001$ ($NPF^{Df/+}$ vs NPF^{sk1}/NPF^{Df}); **e**, $p = 0.0102$ ($NPF^{sk1/+}$ vs NPF^{sk1}/NPF^{Df}), $p = 0.0355$ ($NPF^{Df/+}$ vs NPF^{sk1}/NPF^{Df}); **g**, $p = 0.0337$; **h**, $p = 0.0452$ ($NPF^{sk1/+}$ vs NPF^{sk1}/NPF^{Df}), $p = 0.0034$ ($NPF^{Df/+}$ vs NPF^{sk1}/NPF^{Df}); **i**, $p = 0.0027$ ($NPF^{sk1/+}$ vs NPF^{sk1}/NPF^{Df}), $p = 0.0133$ ($NPF^{Df/+}$ vs NPF^{sk1}/NPF^{Df}).

Supplementary Fig. 2

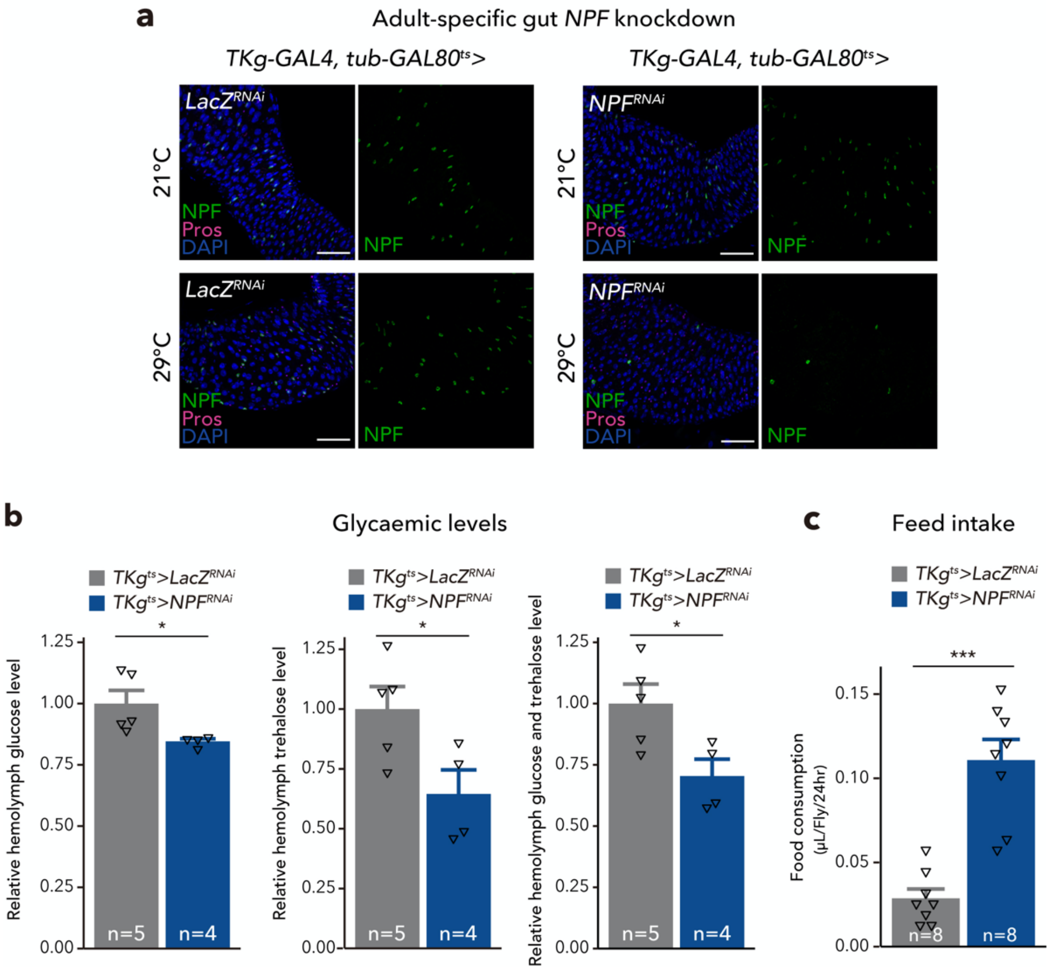

Supplementary Fig. 2. Adult-specific knockdown of *NPF* exhibited similar metabolic phenotypes to *TKg>NPF^{RNAi}*.

a, *TKg-GAL4, tub-GAL80^{ts}*-mediated *NPF* knockdown (*TKg^{ts}>NPF^{RNAi}*) enabled the suppression of *NPF* expression in the gut only in the adult stage. Immunostaining for NPF (green), Prospero (marker for EEC, magenta), and DAPI (blue) in the adult middle midguts. Scale bar, 50 µm. **b**, Relative circulating levels of glucose (left), trehalose (mid), and the sum of glucose and trehalose (right) in *TKg^{ts}>NPF^{RNAi}* animals. The number of samples assessed (n) is indicated in each graph. **c**, Feeding amount measurement for each genotype with the CAFÉ assay. n = 8 samples, each point represents four adult female flies. For RNAi experiments, *LacZ* knockdown (*TKg^{ts}>LacZ^{RNAi}*) was used as negative control. For all bar graphs, mean and SEM with all data points are shown. Statistics: two-tailed Student's *t*-test (b, and c), *$p < 0.05$, **$p < 0.01$, ***$p < 0.001$; NS, non-significant ($p > 0.05$). *p*-values: **b**, (left) $p = 0.0415$, (centre) $p = 0.0377$, (right) $p = 0.0296$; **c**, $p < 0.0001$.

Supplementary Fig. 3

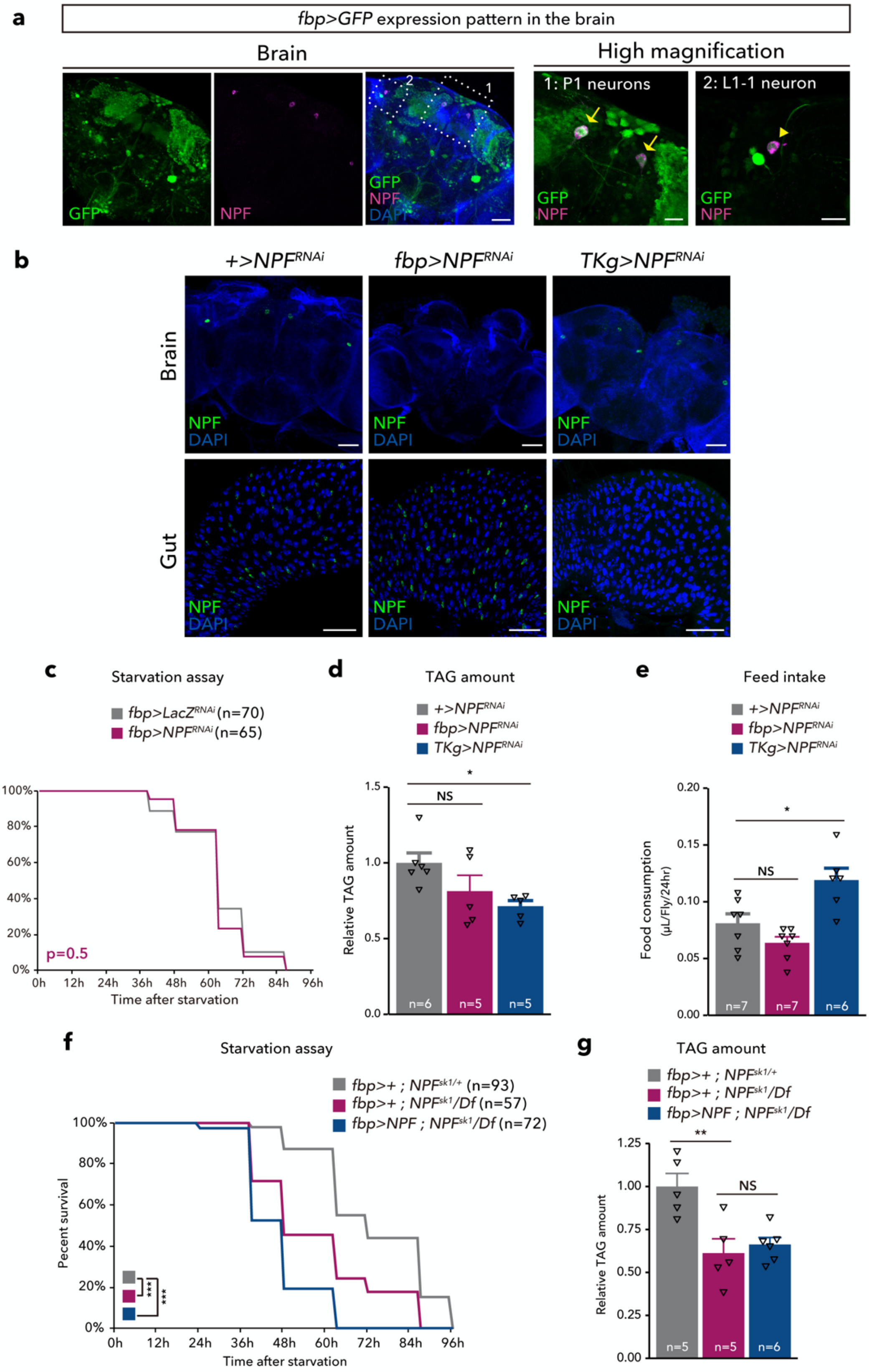

Supplementary Fig. 3. Knockdown of *NPF* in the brain does not reduce lipid storage.

a, (left) Immunofluorescence of the brain in adult flies expressing *UAS-GFP* (green) reporter under *fbp-GAL4* (*fbp>GFP*). Cell bodies of NPF neurons are stained by anti-NPF antibody (magenta). Scale bar, 50 μm. (right) Magnified image of P1 NPF neurons (1), and L1-1 NPF neurons (2). Note that both NPF neurons were co-labelled with *fbp>GFP*. Scale bar, 20 μm. **b**, Immunostaining for NPF (green) and DAPI (blue) in adult brains (top), and gut (bottom) from control (+>*NPF*^{RNAi}), brain-specific *NPF* knockdown animals (*fbp>NPF*^{RNAi}), and gut-specific *NPF* knockdown animals (*TKg>NPF*^{RNAi}). Scale bar, 50 μm. **c**, Survival during starvation in flies of control (*fbp>LacZ*^{RNAi}) and *NPF* knockdown animals in the brain (*fbp>NPF*^{RNAi}). The number of animals assessed (n) is indicated in the graph. **d**, Relative whole-body TAG levels of each genotype. The number of animals assessed (n) is indicated in each graph. **e**, Feeding amount measurement of each genotype with CAFÉ assay. The number of animals assessed (n) is indicated in each graph. Each point contains four adult female flies. **f**, Survival during starvation in flies of each genotype. The number of animals assessed (n) is indicated in each graph. **g**, Relative whole-body TAG levels of each genotype. The number of animals assessed (n) is indicated in each graph. For all bar graphs, mean and SEM with all data points are shown. Statistics: Log rank test with Holm's correction (c, f), one-way ANOVA followed by Tukey's multiple comparisons test (d, e, and g) $^{*}p < 0.05$, $^{**}p < 0.01$, $^{***}p < 0.001$; NS, non-significant ($p > 0.05$). *p*-values: **c**, $p = 0.5000$; **d**, $p = 0.2023$ (+>*NPF*^{RNAi} vs *fbp>NPF*^{RNAi}), $p = 0.0390$ (+>*NPF*^{RNAi} vs *TKg>NPF*^{RNAi}); **e**, $p = 0.2942$ (+>*NPF*^{RNAi} vs *fbp>NPF*^{RNAi}), $p = 0.0121$ (+>*NPF*^{RNAi} vs *TKg>NPF*^{RNAi}); **f**, $p < 0.0001$ (*fbp*>+*; NPF*$^{sk1/+}$ vs *fbp*>+*; NPF*$^{sk1/}$*NPF*^{Df}), $p < 0.0001$ (*fbp*>+*; NPF*$^{sk1/+}$ vs *fbp>NPF; NPF*$^{sk1/}$*NPF*^{Df}); **g**, $p = 0.0040$ (*fbp*>+*; NPF*$^{sk1/+}$ vs *fbp*>+*; NPF*$^{sk1/}$*NPF*^{Df}), $p = 0.8510$ (*fbp*>+*; NPF*$^{sk1/}$*NPF*^{Df} vs *fbp>NPF; NPF*$^{sk1/}$*NPF*^{Df}).

Supplementary Fig. 4

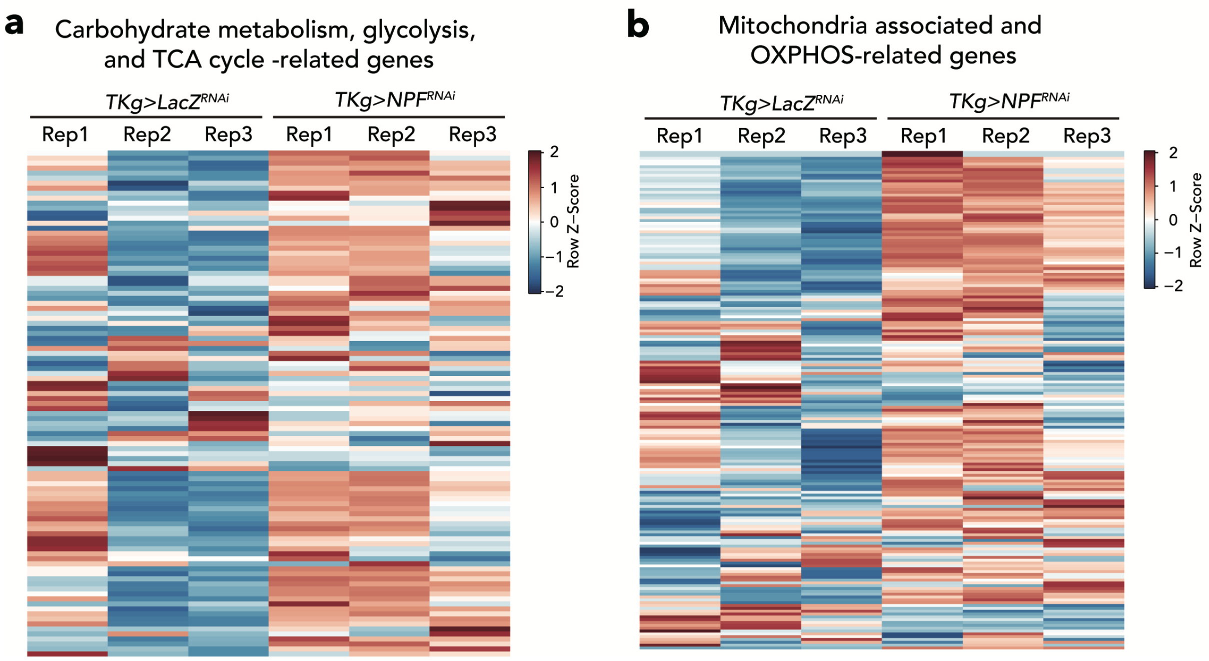

Supplementary Fig. 4. Knockdown of *NPF* in the gut changes carbohydrates and OXPHOS-related gene expression.

a, Expression heatmap of a curated set of metabolism genes showing trend of increase of carbohydrate metabolism, glycolysis, TCA cycle enzyme genes in *TKg>NPF^{RNAi}* animals. Gene expression levels are represented by TMM-normalised FPKM. **b**, Expression heatmap of a curated set of mitochondria-associated genes showing trend of increase expression in *TKg>NPF^{RNAi}* animals. Gene expression levels are represented by TMM-normalised FPKM. Red and blue indicate increased and decreased gene expressions relative to median gene expression levels of *TKg>LacZ^{RNAi}*, respectively; the ratios were plotted on a colour scale (right).

Supplementary Fig. 5

a Cluster hetmap of whole body metabolomics data

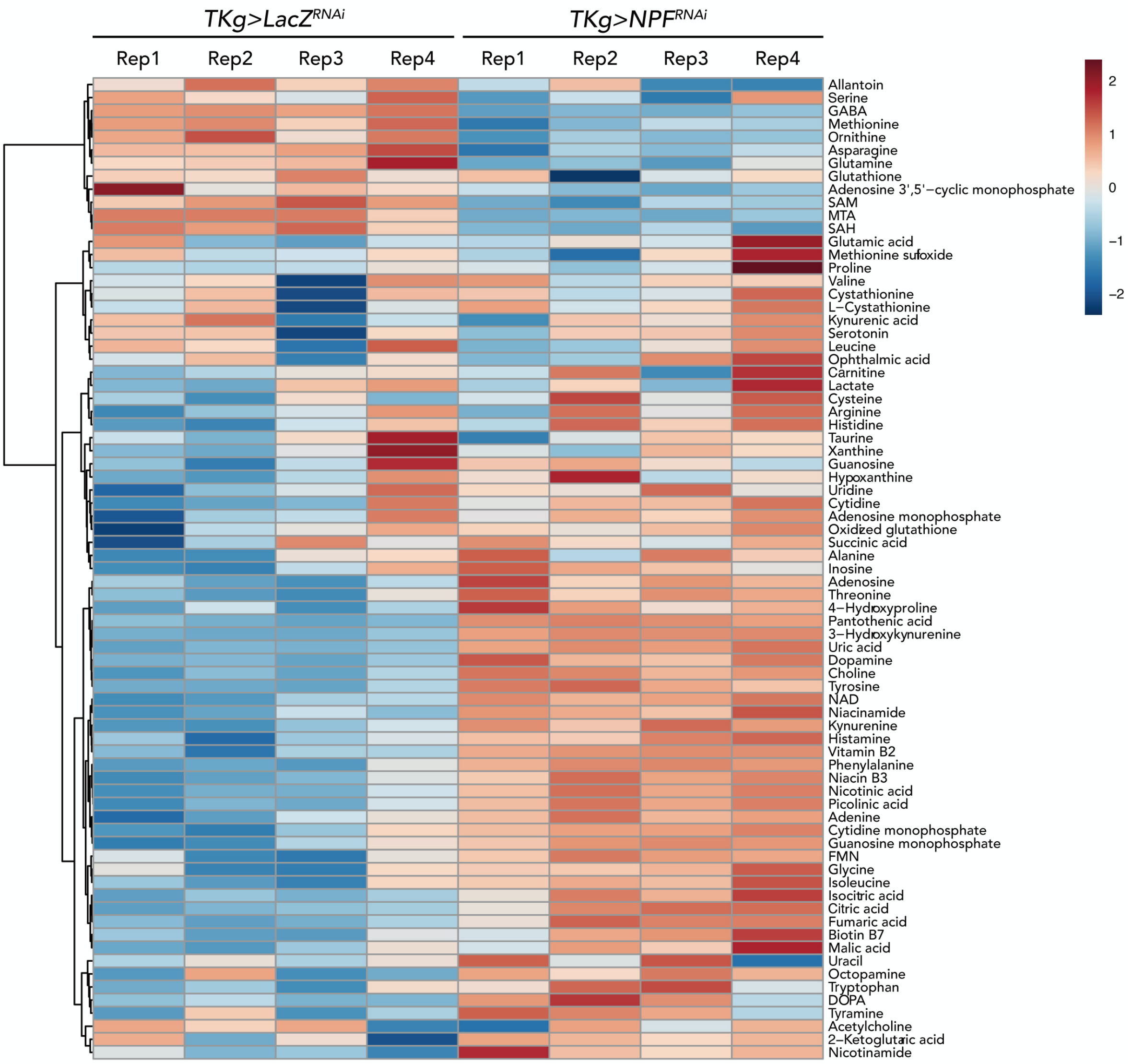

Supplementary Fig. 5. Knockdown of *NPF* in the gut changes metabolite profile of whole-body.

a, Heatmap of clustering of changes of measured whole-body metabolite in *TKg>NPF*^{RNAi} and *TKg>LacZ*^{RNAi}. Note that *NPF* knockdown animals indicated dispersed cluster with control animals. Red and blue indicate increased and decreased metabolites relative to median metabolite levels, respectively; the ratios were plotted on a colour scale (right).

Supplementary Fig. 6

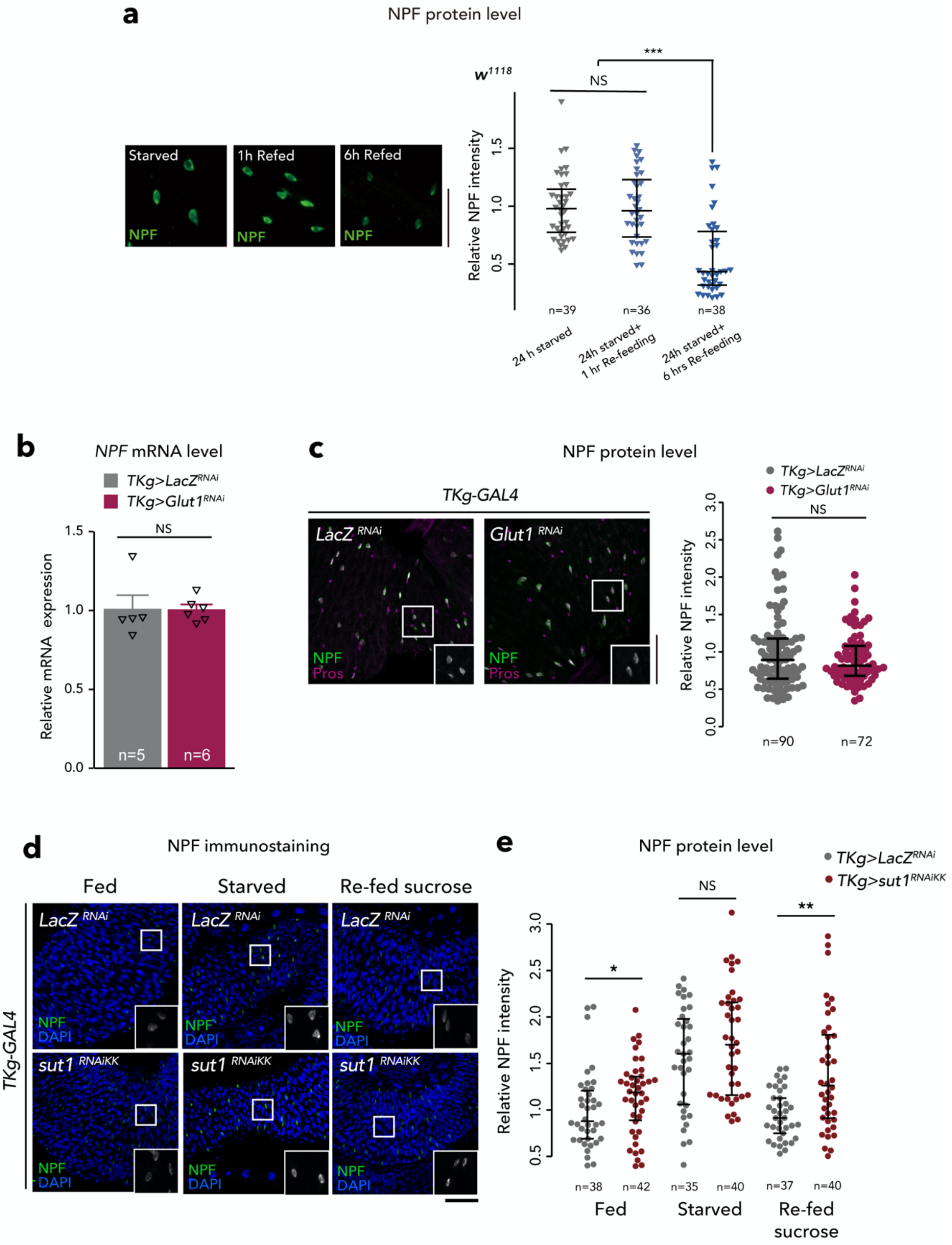

Supplementary Fig. 6. Midgut *NPF* accumulation is restored 6h re-feeding with sucrose.

a, (left) Immunostaining of NPF (green) in adult middle midguts from 6-day-old control (*w^{1118}*) animals starved for 24 h, and fed with sucrose for 1 or 6 h following a 24-h starvation. (right) Quantifications of NPF fluorescent intensity under the condition as in (a). The number of EECs analysed are indicated in the graph. Points of each condition were sampled with more than six guts and correspond to an individual EEC. Scale bar, 25 µm. **b**, RT-qPCR analysis of *NPF* mRNA level following *TKg-GAL4* mediated knockdown of *Glut1* (*TKg>$Glut1^{RNAi}$*). The number of samples assessed (n) is indicated in each graph. **c**, Immunostaining (left) and quantification of florescence (right) for NPF (green/white) and Prospero (magenta) in adult posterior midguts of control (*TKg>$LacZ^{RNAi}$*) and *Glut1* knockdown (*TKg>$Glut1^{RNAi}$*) animals. The number of EECs analysed in each genotype is indicated in the graph. Each point represents NPF fluorescent intensity in a single EEC. For each genotype, more than seven guts. Scale bar, 50 µm. **d**, Immunostaining for NPF (green/white) and DAPI (blue) in adult middle midguts from 6-day-old control *TKg>$lacZ^{RNAi}$* and *TKg>$sut1^{RNAi}$* animals fully fed (Fed), on 48 h starvation (Starved), and on re-feeding with sucrose following a 24 h starvation period (Re-fed sucrose). Scale bar, 50 µm. **e**, Quantifications of NPF fluorescent intensity of each genotype as in (d). The number of EECs analysed (n) are indicated in the graph. Each point represents NPF fluorescent intensity in a single EEC. For each genotype, more than eight guts were used. For all bar graphs, mean and SEM with all data points are shown. For all dot blots, the three horizontal lines on each graph indicate lower, median, and upper quartiles. Statistics: Wilcoxon rank sum test with Holm' correction (a, c, and e), two-tailed Student's *t*-test (b). *$p < 0.05$, **$p < 0.01$, ***$p < 0.001$; NS, non-significant ($p > 0.05$). *p*-values: **a**, $p = 0.99$ (Starved vs 1h Re-feeding), $p < 0.0001$ (Starved vs 6h Re-feeding), $p < 0.0001$ (1h Re-feeding vs 6h Re-feeding); **b**, $p = 0.9715$; **c**, $p = 0.7171$; **e**, $p = 0.0442$ (left), $p = 0.2597$ (centre), $p = 0.0011$ (right).

Supplementary Fig. 7

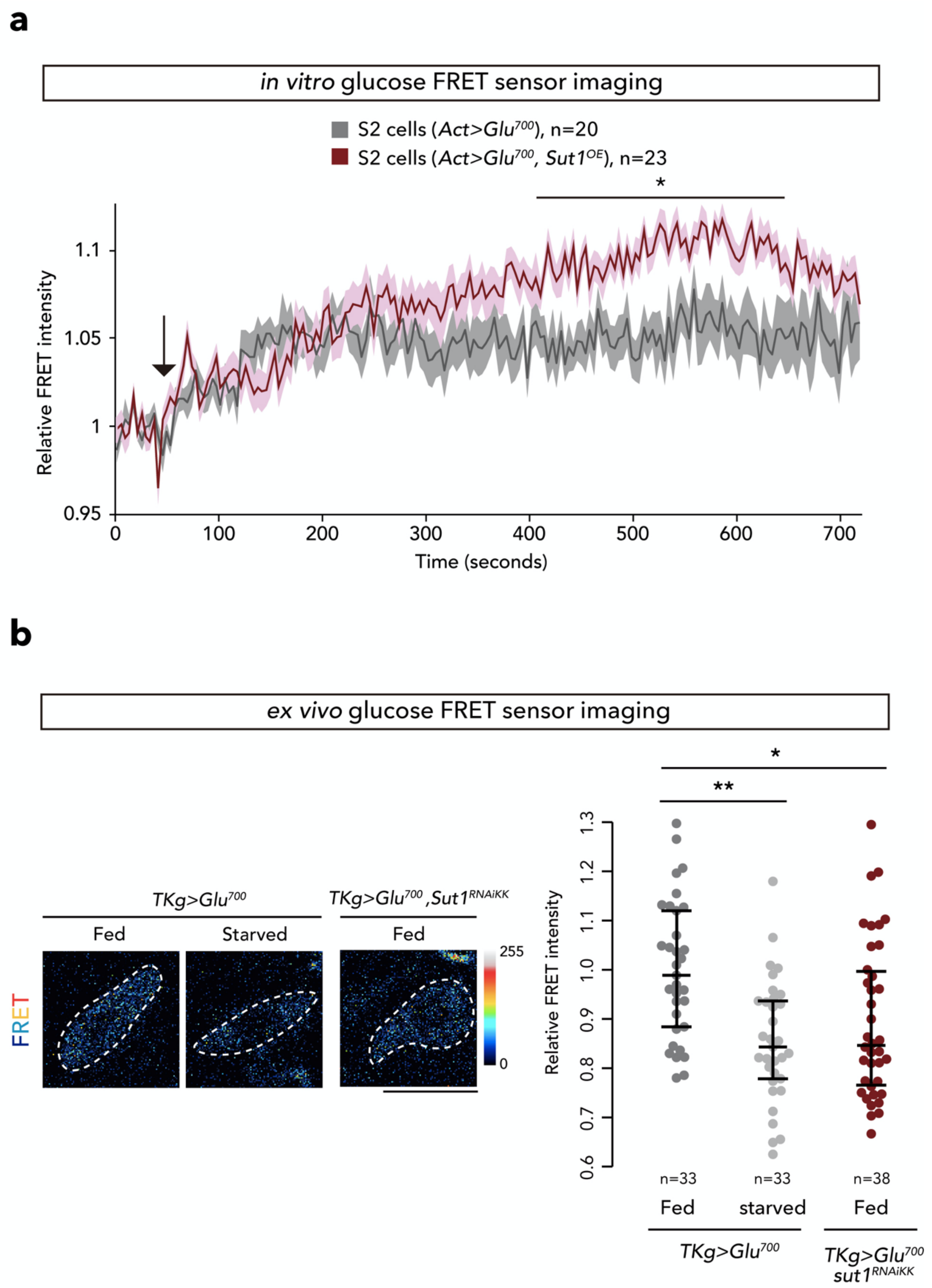

Supplementary Fig. 7. Sut1 in the EECs regulates intracellular glucose level.

a, Changes in the relative fluorescence intensity of Glu^{700} glucose sensor FRET (YFP/CFP) signal of *Act>Glu^{700}* S2 cells (grey), and *Act>Glu^{700}, sut1^{OE}* S2 cells (red) after 750 s exposure

to glucose solution (final conc. 25 mM). Arrow indicates the time (40 s) of glucose administration. Note that *sut1* overexpression significantly increased the FRET signal in response to glucose administration between 400 s and 650 s. Statistical analysis was performed with average FRET levels from 400 s to 650 s of each genotype. The number of samples assessed (n) are indicated in the graphs. Data are presented as mean values ± SEM. **b**, Glu^{700} glucose sensor FRET signal in EECs of ad libitum feeding control (*TKg>Glu^{700}*), 24 h starved control, and ad libitum feeding *sut1* knockdown (*TKg>Glu^{700}, $sut1^{RNAiKK}$*) animals. (Left) Representative FRET image in EECs (dashed lines). Scale bar: 10 µm. Fluorescence signals are pseudocoloured; high (Max: 255) to low (Minimum: 0) intensity is displayed with a colour scale. (Right) Quantification of FRET signals. Median FRET ratio for each genotype was set at 1 for ad libitum feeding control. The number of samples assessed (n) are indicated in the graphs. Data are presented with three horizontal lines indicating lower, median, and upper quartiles. Statistics: Wilcoxon rank sum test with Holm' correction (a, and b). $*p < 0.05$, $**p < 0.01$; NS, non-significant ($p > 0.05$). *p*-values: **a**, $p < 0.0001$; **b**, $p = 0.0002$ (*TKg>Glu^{700}* Fed vs *TKg>Glu^{700}* Starved), $p = 0.0002$ (*TKg>Glu^{700}* Fed vs *TKg>Glu^{700}, $sut1^{RNAi}$* Fed).

Supplementary Fig. 8

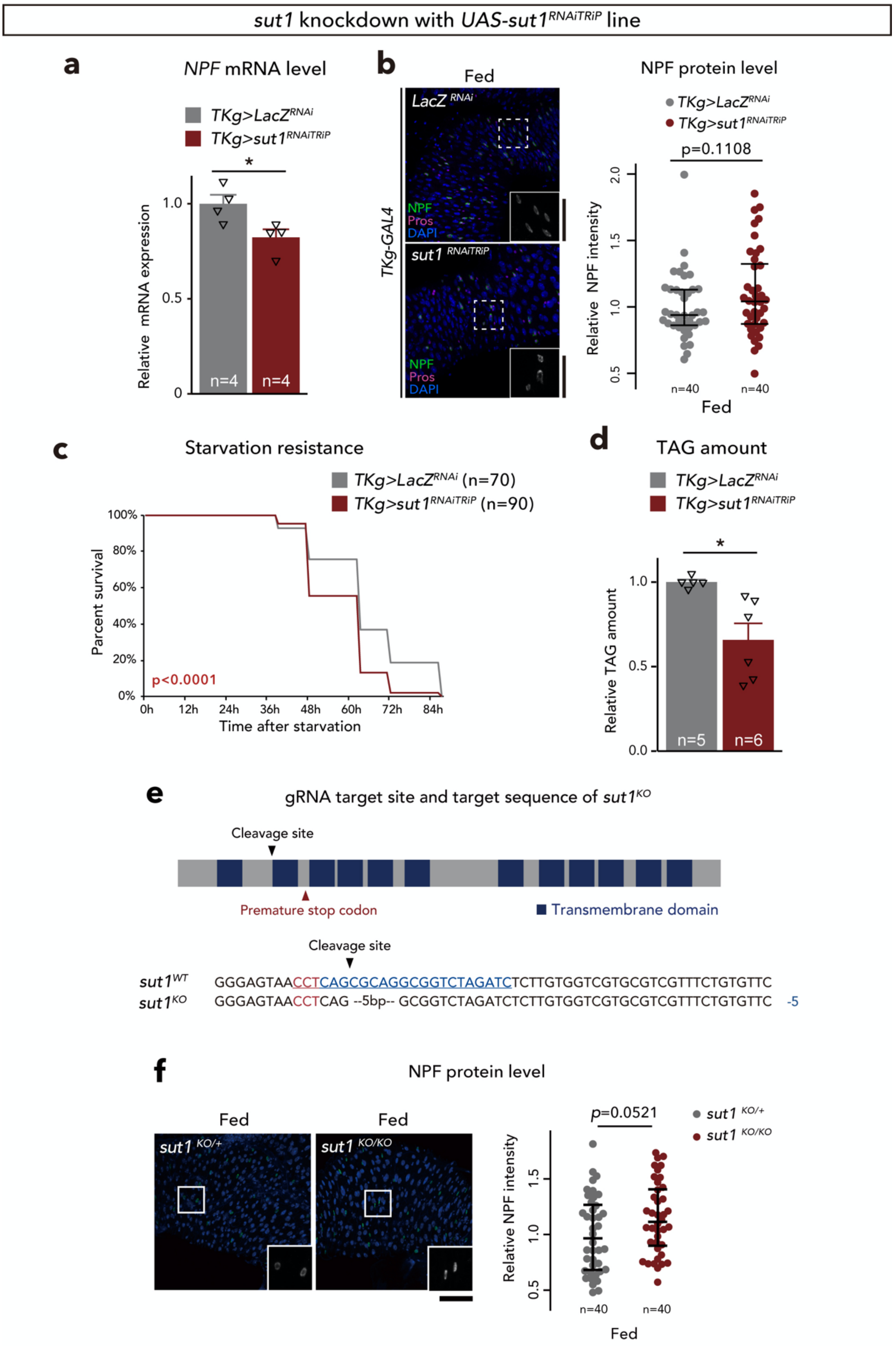

Supplementary Fig. 8. Sut1 in the EECs regulates NPF production.

a, RT-qPCR analysis of *NPF* mRNA level following *TKg-GAL4* mediated knockdown of *sut1* with another *UAS-sut1*^{RNAi} line (*TKg>sut1*^{RNAiTRiP}). The number of samples assessed (n) is indicated in the graph. **b**, (left) Immunostaining for NPF (green/white), Prospero (magenta), and DAPI (blue) in adult middle midguts from *TKg>sut1*^{RNAiTRiP} animals. Scale bar, 50 µm. (right) Quantifications of NPF fluorescent intensity for each genotype. The number of EECs analysed in each genotype is indicated in the graph. Each point represents NPF fluorescent intensity in a single EEC. For each genotype, more than eight guts were used. **c**, Survival during starvation in *TKg>sut1*^{RNAiTRiP} flies. The number of animals assessed (n) is indicated in each graph. **d**, Relative whole-body TAG levels. The number of samples assessed (n) is indicated in each graph. **e**, A schematic representation of sut1 coding sequence structure, gRNA target sequence (blue), and deletion of *sut1* knockout (*sut1*^{KO}) mutant allele. Regions of the putative transmembrane domains of Sut1 are highlighted in dark blue. A premature stop codon generated in the *sut1*^{KO} allele is indicated by a red arrowhead. DNA sequences of wild-type (*WT*) and *sut1*^{KO} alleles are shown. The Cas9-gRNA target sequence is underlined. The PAM sequence is indicated in red. The 5 bp deletion results in a premature stop codon between the second and third transmembrane domains of Sut1. **f**, (left) Immunostaining for NPF (green/white) and DAPI (blue) in adult middle midguts from *sut1* mutant animals. Scale bar, 50 µm. (right) Quantification of NPF fluorescent intensity for each genotype as described in (e). The number of EECs analysed in each genotype is indicated in the graph. Each point represents NPF fluorescent intensity in a single EEC. For each genotype, more than eight guts were used. For all bar graphs, mean and SEM with all data points are shown. For all dot blots, the three horizontal lines on each graph indicate lower, median, and upper quartiles. Statistics: two-tailed Student's *t*-test (a, and d), Wilcoxon rank sum test (b, and f), Log rank test (c). *$p < 0.05$; NS, non-significant ($p > 0.05$). *p*-values: **a**, $p = 0.0316$; **b**, $p = 0.1108$; **c**, $p < 0.0001$; **d**, $p = 0.0117$; **e**, $p = 0.0521$.

Supplementary Fig. 9

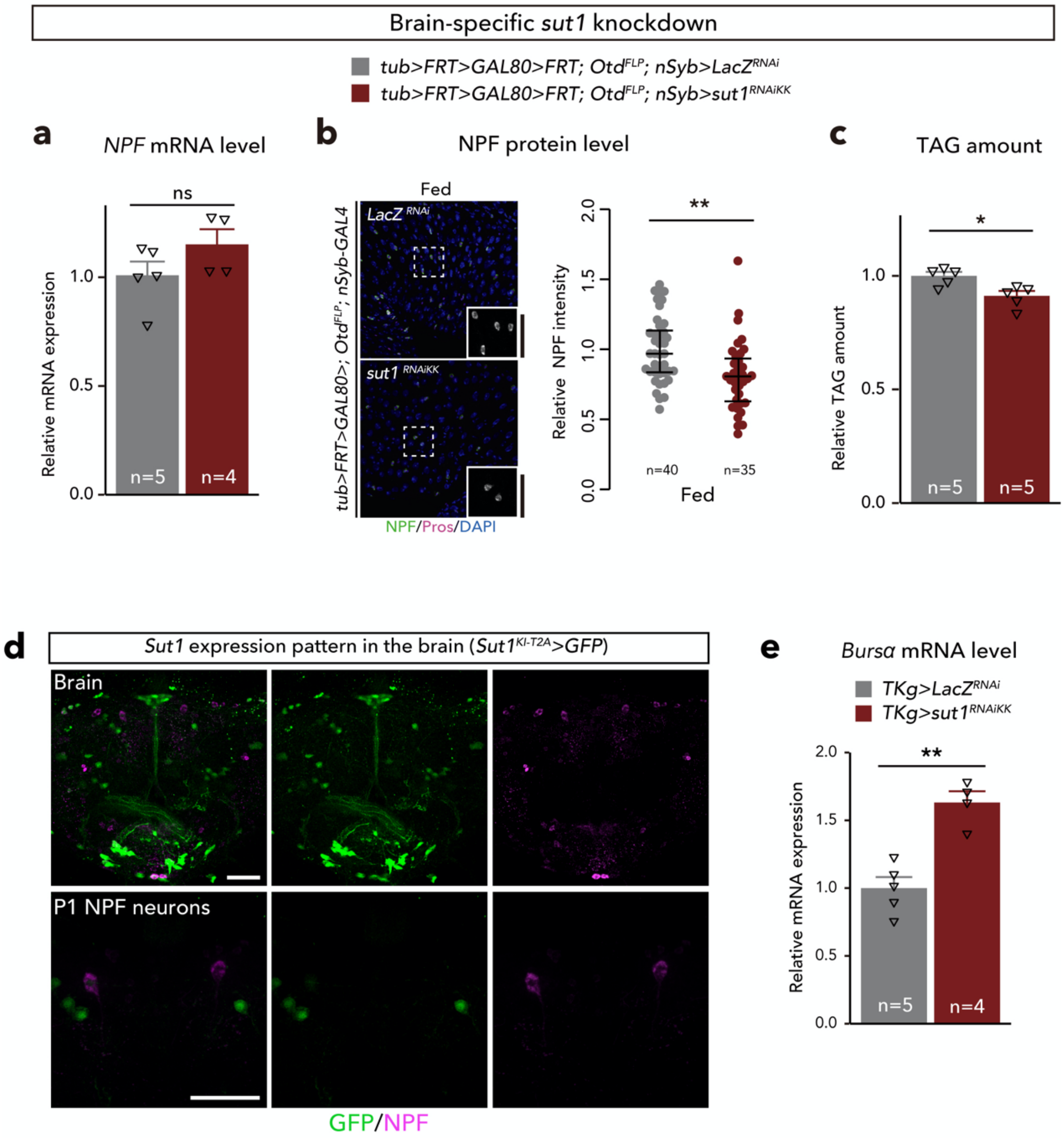

Supplementary Fig. 9. *sut1* knockdown in the brain does not attenuate gut NPF.

a, RT-qPCR analysis of *NPF* mRNA level in brain-specific *sut1* knockdown flies using *Otd-FLP* flies. The number of samples assessed (n) is indicated in the graph. **b**, (left) Immunostaining for NPF (green/white), Prospero (magenta), and DAPI (blue) in adult middle midguts from brain-specific *sut1* knockdown animals. Scale bar, 50 µm. (right) Quantification of NPF fluorescent intensity for each genotype. The number of EECs analysed in each genotype is indicated in the graph. Each point represents NPF fluorescent intensity in a single EEC. For each genotype, more than eight guts were used. **c**, Relative whole-body TAG levels. The number of samples assessed (n) is indicated in each graph. **d**, Immunofluorescence of *sut1*$^{KI\text{-}T2A}$*-GAL4-*

driven *UAS-GFP* in the brain (top), and P1 NPF neurons (bottom) stained for GFP (green), and NPF (magenta). Note, no NPF^{+} neurons exhibiting $sut1^{KI\text{-}T2A}$-*GAL4*-driven GFP signals were detected. Scale bar, 50 μm. **e**, RT-qPCR analysis of *Bursα* mRNA level in *TKg>*$sut1^{RNAi}$ guts. The number of samples assessed (n) is indicated in the graph. For RNAi experiments, *LacZ* knockdown was used as negative control. For all bar graphs, mean and SEM with all data points are shown. For dot blot, the three horizontal lines on each graph indicate lower, median, and upper quartiles. Statistics: two-tailed Student's *t*-test (a, c, and e), Wilcoxon rank sum test (b). *p < 0.05, **p < 0.01; NS, non-significant (p > 0.05). p-values: **a**, p = 0.1766; **b**, p = 0.0008; **c**, p = 0.0138; **d**, p = 0.0011.

Supplementary Fig. 10

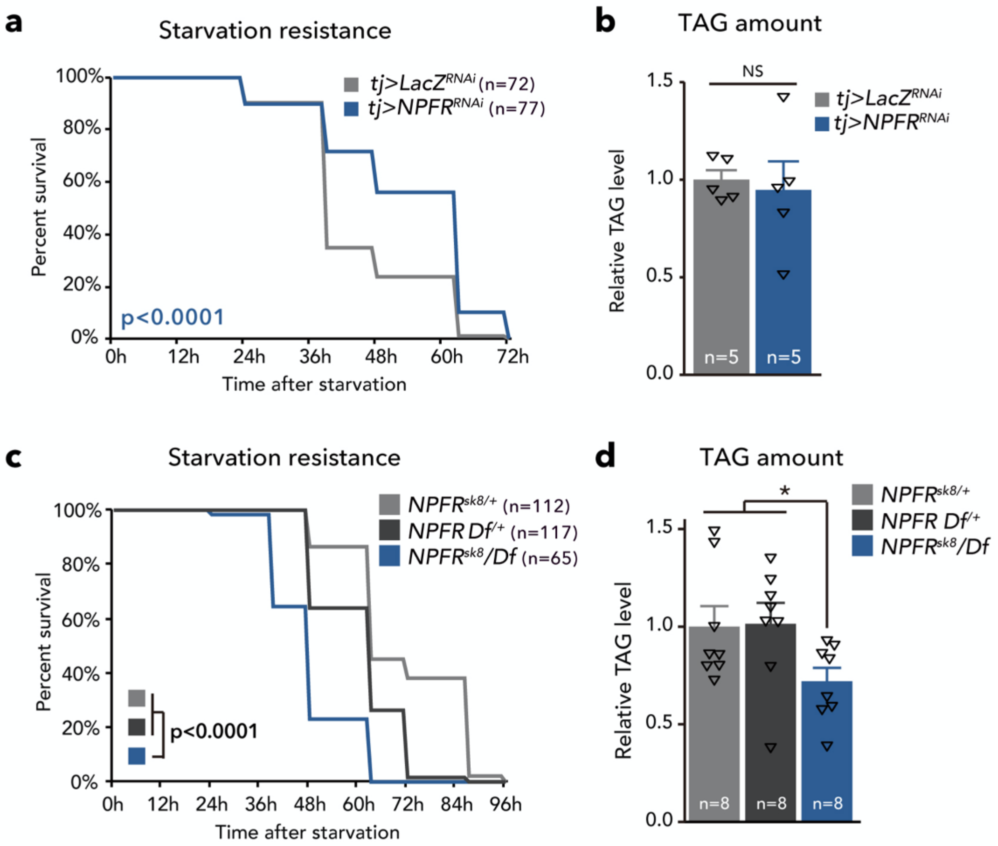

Supplementary Fig. 10. NPFR in the ovarian somatic cell does not affect lipid storage.

a, Survival during starvation in flies of control (*tj>LacZ*^{RNAi}) and *NPFR* knockdown animals in the ovarian somatic cells (*tj>NPFR*^{RNAi}). The number of animals assessed (n) is indicated in the graphs. **b**, Relative whole-body TAG levels of each genotype as in (a). The number of samples assessed (n) is indicated in the graphs. **c**, Survival during starvation in flies of control (*NPFR*$^{sk8/+}$ and *NPFR*$^{Df/+}$), and *NPFR*$^{sk8/Df}$. The number of animals assessed (n) is indicated in the graphs. **d**, Relative whole-body TAG levels of each genotype as in (d). The number of samples assessed (n) is indicated in the graphs. For all bar graphs, mean and SEM with all data points are shown. Statistics: Log rank test with Holm's correction (a, and c), two-tailed Student's *t*-test (b), two-tailed Student's *t*-test with Holm's correction (d). $*p < 0.05$; NS, non-significant ($p > 0.05$). *p*-values: **a**, $p < 0.0001$; **b**, $p = 0.7409$; **c**, $p < 0.0001$ (*NPFR*$^{sk8/+}$ vs *NPFR*$^{sk8/}$*NPFR*^{Df}), $p < 0.0001$ (*NPFR*$^{Df/+}$ vs *NPFR*$^{sk8/}$*NPFR*^{Df}); **d**, $p = 0.0314$ (*NPFR*$^{sk8/+}$ vs *NPFR*$^{sk8/}$*NPFR*^{Df}), $p = 0.0291$ (*NPFR*$^{Df/+}$ vs *NPFR*$^{sk8/}$*NPFR*^{Df}).

Supplementary Fig. 11

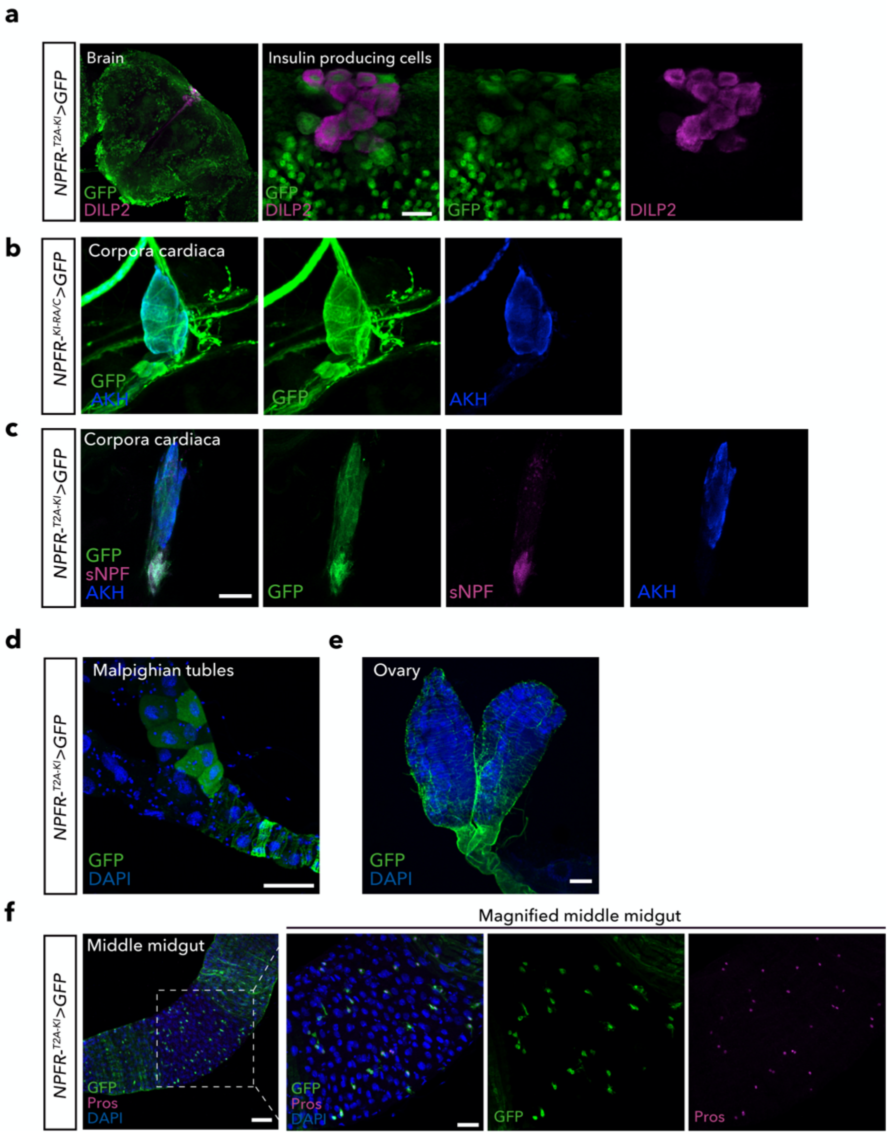

Supplementary Fig. 11. Expression pattern of *NPFR* knock-in *GAL4* lines.
a, Immunofluorescence of the brain in adult flies expressing *UAS-GFP* (green) reporter under *NPFR*$^{KI\text{-}T2A}$*-GAL4*. Cell bodies of insulin-producing cells are stained by anti-DILP2 antibody (magenta). Scale bar, 20 μm. **b**, **c**, Immunofluorescence of the corpora cardiaca (CC) in adult flies expressing *UAS-GFP* (green) reporter under two *NPFR*^{KI}*-GAL4* lines. Cell bodies of sNPF neurons and CC are stained by anti-sNPF antibody (magenta) and anti-AKH antibody (blue), respectively. Scale bar, 20 μm. **d**, Immunofluorescence of the Malpighian tubules in adult flies expressing *UAS-GFP* (green) reporter under *NPFR*$^{KI\text{-}T2A}$*-GAL4*. Scale bar, 50 μm. **e**, Immunofluorescence of the ovary in adult flies expressing *UAS-GFP* (green) reporter under *NPFR*$^{KI\text{-}T2A}$*-GAL4*. Scale bar, 100 μm. **e**, Immunofluorescence of the middle midgut in adult flies expressing *UAS-GFP* (green) reporter under *NPFR*$^{KI\text{-}T2A}$*-GAL4*. Note that several GFP+ cells are labelled by the EEC marker Prospero (magenta). Scale bar, (left) 50 μm, (right) 25 μm.

Supplementary Fig. 12

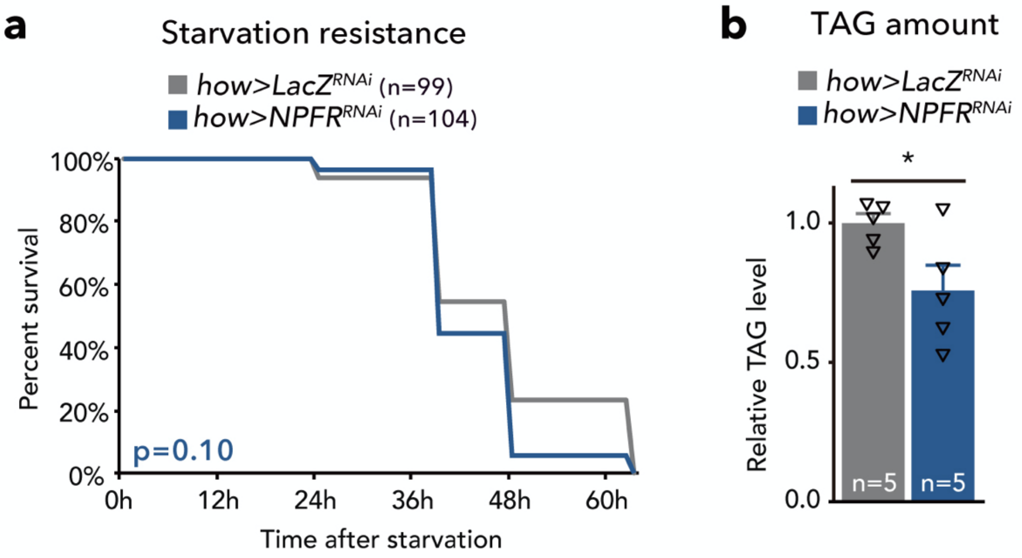

Supplementary Fig. 12. NPFR in the visceral muscle has small effect on starvation resistance and lipid amount.

a, Survival during starvation in flies of control (*how>LacZ*^{RNAi}), and *NPFR* knockdown animals in the visceral muscle (*how>NPFR*^{RNAi}). The number of animals assessed (n) is indicated in the graphs. **b**, Relative whole-body TAG levels of each genotype. The number of samples assessed (n) is indicated in the graphs. For bar graph, mean and SEM with all data points are shown. Statistics: Log rank test (g), two-tailed Student's *t*-test (h). *$p < 0.05$; NS, non-significant ($p > 0.05$). *p*-values: **a**, $p = 0.1000$; **b**, $p = 0.0364$.

Supplementary Fig. 13

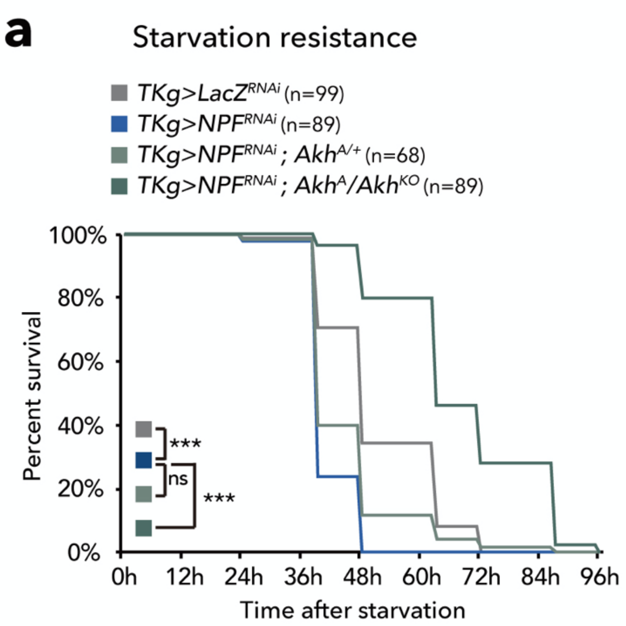

Supplementary Fig. 13. Loss of AKH restores starvation resistance and lipid reduction of *TKg>NPF^{RNAi}*.

a, Survival during starvation in flies of each genotype. The number of animals assessed (n) is indicated in the graphs. **b**, Relative whole-body TAG levels of each genotype. The number of animals assessed (n) is indicated in the graphs. For all bar graphs, mean and SEM with all data points are shown. Statistics: Log rank test with Holm's correction (a), one-way ANOVA followed by Tukey's multiple comparisons test (b). ***p* < 0.01, ****p* < 0.001; NS, non-significant (*p* > 0.05). *p*-values: **a**, *p* < 0.0001 (*TKg>LacZ^{RNAi}* vs *TKg>NPF^{RNAi}*), *p* = 0.0765 (*TKg> NPF^{RNAi}; Akh$^{A/+}$* vs *TKg>NPF^{RNAi}*), *p* < 0.0001 (*TKg> NPF^{RNAi}; Akh$^{A/}$Akh^{KO}* vs *TKg>NPF^{RNAi}*); **b**, *p* = 0.0014 (*TKg>LacZ^{RNAi}* vs *TKg>NPF^{RNAi}*), *p* = 0.3028 (*TKg> NPF^{RNAi}; Akh$^{A/+}$* vs *TKg>NPF^{RNAi}*), *p* < 0.0001 (*TKg> NPF^{RNAi}; Akh$^{A/}$Akh^{KO}* vs *TKg>NPF^{RNAi}*).

Supplementary Fig. 14

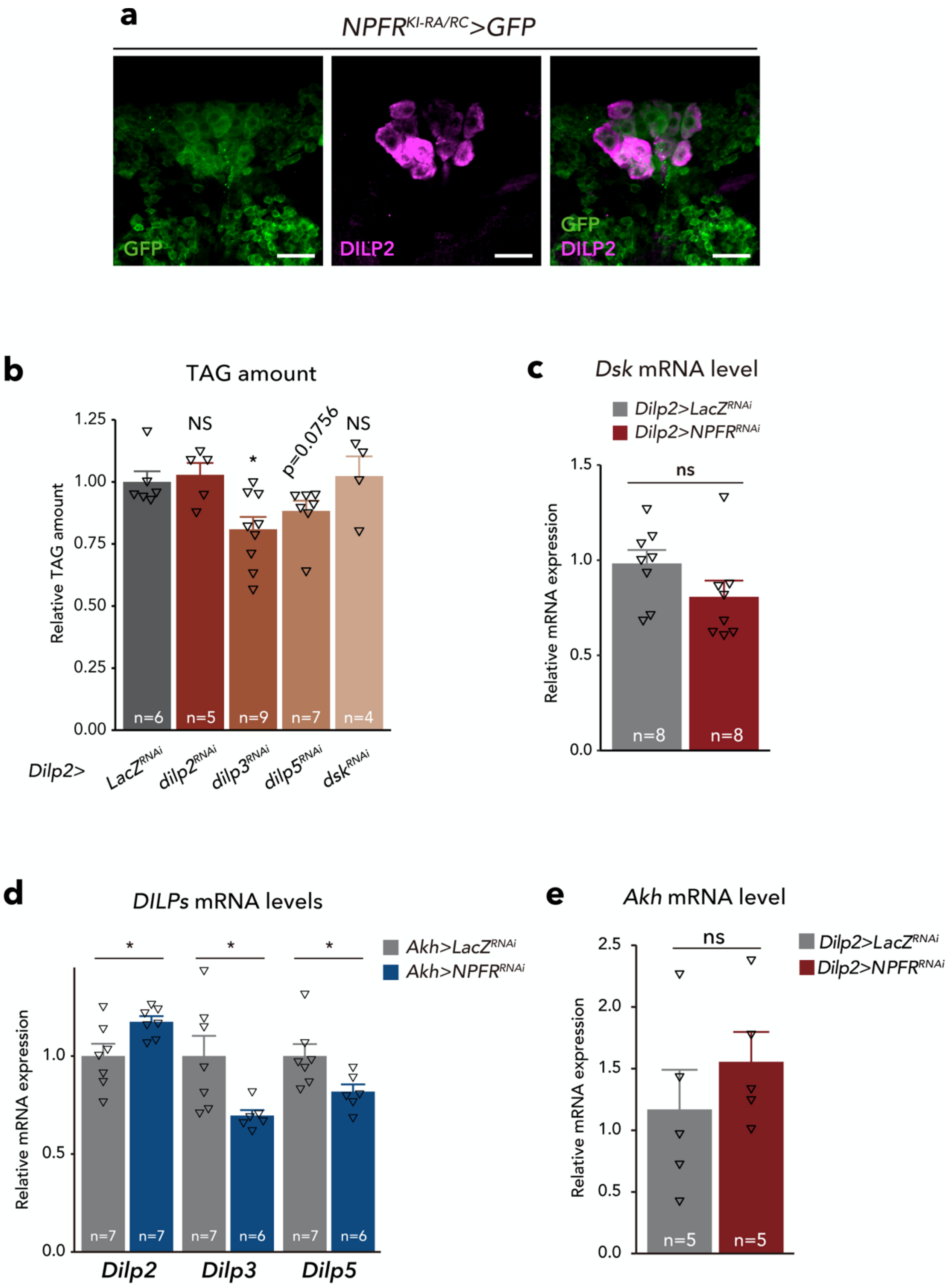

Supplementary Fig. 14. NPF neurons does not have direct connection with the IPCs.

a, Immunofluorescence of the IPCs in adult flies expressing *UAS-mCD8::GFP* (green) reporter under *NPFR*$^{KI\text{-}RA/RC}$*-GAL4*. Cell bodies of IPCs are stained by anti-DILP2 (magenta). Scale bar, 20 μm. **b**, Relative whole-body TAG levels of each genotype. The number of

samples assessed (n) is indicated in the graphs. **c**, RT-qPCR analysis of *Dsk* mRNA level in *Dilp2>NPFR*^{RNAi} flies. The number of samples assessed (n) is indicated in the graph. **d**, RT-qPCR analysis of *dilps* mRNA level in CC-specific *NPFR* knockdown flies (*Akh>NPFR*^{RNAi}). The number of samples assessed (n) is indicated in the graph. **e**, RT-qPCR analysis of *Akh* mRNA level in IPC-specific *NPFR* knockdown flies (*Dilp2>NPFR*^{RNAi}). The number of samples assessed (n) is indicated in the graph. For RNAi experiments, *LacZ* knockdown was used as negative control. For all bar graphs, mean and SEM with all data points are shown. Statistics: two-tailed Student's *t*-test (b-e). *$p < 0.05$; NS, non-significant ($p > 0.05$). *p*-values; **b**, $p = 0.6658$ (*Dilp2>LacZ*^{RNAi} vs *Dilp2>dilp2*^{RNAi}), $p = 0.0182$ (*Dilp2>LacZ*^{RNAi} vs *Dilp2>dilp3*^{RNAi}), $p = 0.0757$ (*Dilp2>LacZ*^{RNAi} vs *Dilp2>dilp5*^{RNAi}), $p = 0.7835$ (*Dilp2>LacZ*^{RNAi} vs *Dilp2>dsk*^{RNAi}); **c**, $p = 0.1364$; **d**, $p = 0.0260$ (*dilp2*), $p = 0.0227$ (*dilp3*), $p = 0.0324$ (*dilp5*); **e**, $p = 0.3645$.

Supplementary Fig. 15

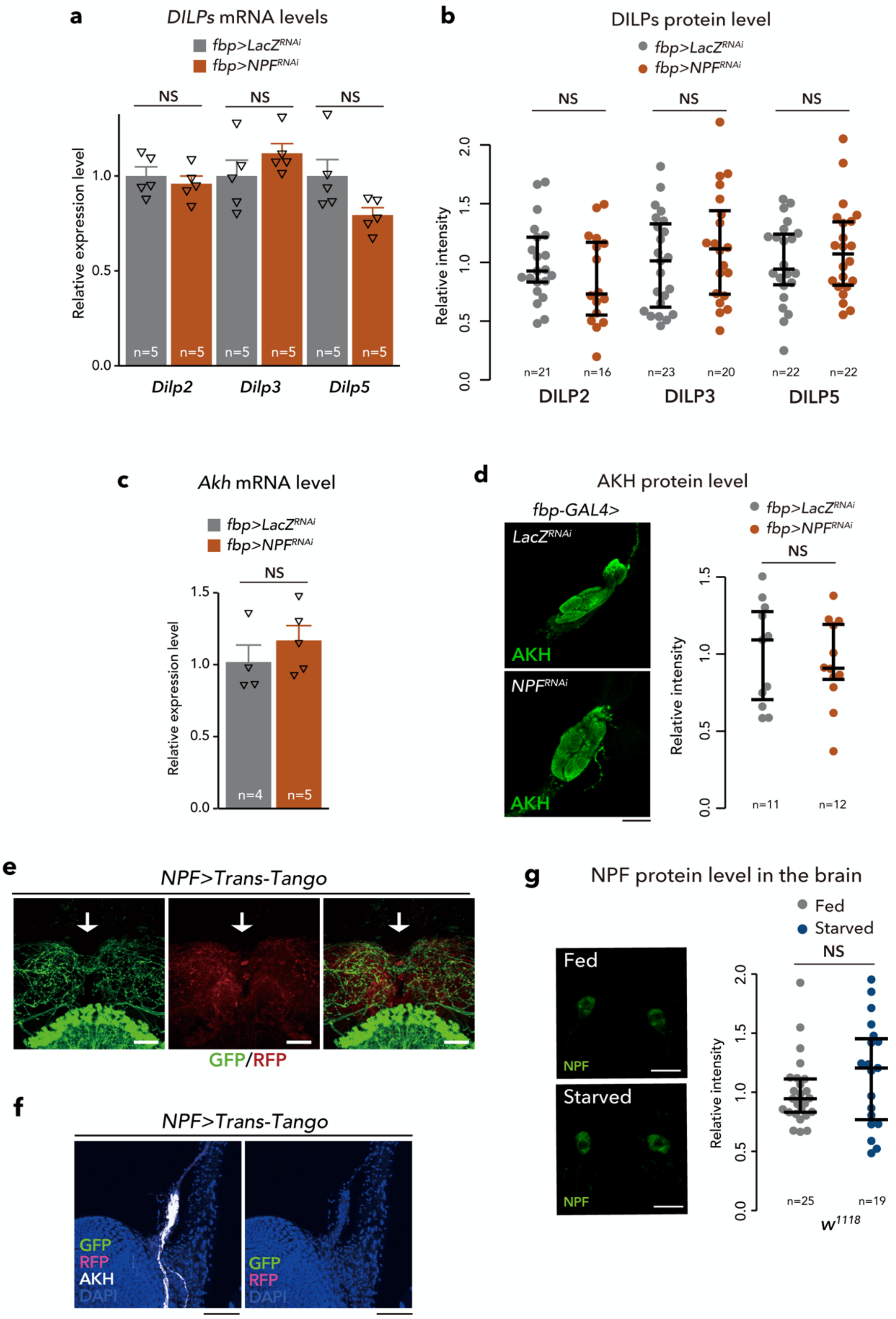

Supplementary Fig. 15. Brain NPF does not affect the levels of DILPs and AKH.

a, RT-qPCR analysis of *dilps* mRNA level in brain-specific *NPF* knockdown flies (*fbp>NPF*^{RNAi}). The number of samples assessed (n) is indicated in the graph. **b**, Quantification of DILP2, 3, and 5 in the brain of adult *fbp>NPF*^{RNAi} animals. Scale bar, 20 μm. The number of samples assessed (n) is indicated in the graph. **c**, RT-qPCR analysis of *Akh* mRNA levels in the whole bodies of *fbp>NPF*^{RNAi} adult animals. The number of samples assessed (n) is indicated in the graph. **d**, Quantification of AKH (green) in the CC of *fbp>NPF*^{RNAi} adult flies. Scale bar, 10 μm. The number of samples assessed (n) is indicated in the graph. **e**, Immunofluorescence of the PI region (arrow) in adult flies expressing *Trans-Tango* driven by *NPF-GAL4* (*NPF>Trans-Tango*). Scale bar, 20 μm. Note, postsynaptic signal (RFP; red) was not observed in the PI region. **f**, Immunofluorescence of the CC in *NPF>Trans-Tango* adult flies. Cell bodies of CC are stained with anti-AKH antibody (white). Scale bar, 10 μm. Note, neither presynaptic signals (GFP; green) nor postsynaptic signals (RFP; magenta) were observed near the CC. **g**, (left) Immunostaining for NPF (green) of P1 NPF neurons in the adult brains of ad libitum feeding, and 24h starved w^{1118} flies. Scale bar, 20 μm. (right) Quantification of NPF fluorescent intensity. The number of samples assessed (n) is indicated in the graph. For RNAi experiments, *LacZ* knockdown (*fbp>lacZ*^{RNAi}) was used as negative control. For all bar graphs, mean and SEM with all data points are shown. For all dot blots, the three horizontal lines on each graph indicate lower, median, and upper quartiles. Statistics: two-tailed Student's *t*-test (a, and c), Wilcoxon rank sum test (b, d, and g), NS, non-significant ($p > 0.05$). *p*-values: **a**, $p = 0.5302$ (*dilp2*), $p = 0.2543$ (*dilp3*), $p = 0.0617$ (*dilp5*); **b**, $p = 0.2411$ (DILP2), $p = 0.3779$ (DILP3), $p = 0.7187$ (DILP5); **c**, $p = 0.3655$; **d**, $p = 0.8328$; **g**, $p = 0.2685$.

Supplementary Data Legends

Supplementary Data 1. FPKM values of carbohydrate metabolism-related gene expression in the abdomens from *TKg>LacZ^{RNAi}* and *TKg>NPF^{RNAi}* virgin females.
Supplementary Figure 4a was created based on these data. We analyzed three independent samples of each genotype.

Supplementary Data 2. FPKM values of mitochondria-related gene expression in the abdomens from *TKg>LacZ^{RNAi}* and *TKg>NPF^{RNAi}* virgin females.
Supplementary Figure 4b was created based on these data. We analyzed three independent samples of each genotype.

Supplementary Data 3. Amount (pmol/mg body weight) of metabolites in whole body samples from *TKg>LacZ^{RNAi}* and *TKg>NPF^{RNAi}* virgin females.
Figures 2a, 2b, 2c and Supplementary Figure 5a were created based on these data. We analyzed four independent samples of each genotype.

Supplementary Data 4. Amount (nmol/mg protein in the hemolymph) of metabolites in hemolymph samples from *TKg>LacZ^{RNAi}* and *TKg>NPF^{RNAi}* virgin females.
Figures 2a, 2b, and 2c were created based on these data. We analyzed four independent samples of each genotype.

Supplementary Data 5. FPKM values of starvation-induced gene expression in the abdomens from *TKg>LacZ^{RNAi}* and *TKg>NPF^{RNAi}* virgin females.
Figure 2d was created based on these data. We analyzed three independent samples of each genotype.

Supplementary Data 6. Primers used in this study.
Primer names and their sequences (5´ > 3´) are described.
