# Supplementary material for: The sugar-responsive enteroendocrine neuropeptide F regulates lipid metabolism through glucagon-like and insulin-like hormones in Drosophila melanogaster
Source: Nat Commun. 2021 Aug 10;12:4818. doi: 10.1038/s41467-021-25146-w (PMC8355161; doi:10.1038/s41467-021-25146-w)
Supplement: Supplementary file 9 — Description of additional supplementary files [file 41467_2021_25146_MOESM9_ESM.docx]

Description of additional supplementary files

Title: Supplementary Data 1.

Description: FPKM values of carbohydrate metabolism-related gene expression in the abdomens from TKg>LacZRNAi and TKg>NPFRNAi virgin females. Supplementary Figure 4a was created based on these data. We analyzed three independent samples of each genotype.

Title: Supplementary Data 2.

Description: FPKM values of mitochondria-related gene expression in the abdomens from TKg>LacZ RNAi and TKg>NPFRNAi virgin females. Supplementary Figure 4b was created based on these data. We analyzed three independent samples of each genotype.

Title: Supplementary Data 3.

Description: Amount (pmol/mg body weight) of metabolites in whole body samples from TKg>LacZRNAi and TKg>NPFRNAi virgin females. Figures 2a, 2b, 2c and Supplementary Figure 5a were created based on these data. We analyzed four independent samples of each genotype.

Title: Supplementary Data 4.

Description: Amount (nmol/mg protein in the hemolymph) of metabolites in hemolymph samples from TKg>LacZRNAi and TKg>NPFRNAi virgin females. Figures 2a, 2b, and 2c were created based on these data. We analyzed four independent samples of each genotype.

Title: Supplementary Data 5.

Description: FPKM values of starvation-induced gene expression in the abdomens from TKg>LacZRNAi and TKg>NPFRNAi virgin females. Figure 2d was created based on these data. We analyzed three independent samples of each genotype.

Title: Supplementary Data 6.

Description:Primers used in this study. Primer names and their sequences (5´ > 3´) are described.
