# Supplementary material for: The sugar-responsive enteroendocrine neuropeptide F regulates lipid metabolism through glucagon-like and insulin-like hormones in Drosophila melanogaster
Source: Nat Commun. 2021 Aug 10;12:4818. doi: 10.1038/s41467-021-25146-w (PMC8355161; doi:10.1038/s41467-021-25146-w)
Supplement: Supplementary file 10 — Reporting Summary [file 41467_2021_25146_MOESM10_ESM.pdf]

nature research

Corresponding author(s): Ryusuke Niwa

Last updated by author(s): Jul 7, 2021

Reporting Summary

Nature Research wishes to improve the reproducibility of the work that we publish. This form provides structure for consistency and transparency in reporting. For further information on Nature Research policies, see our Editorial Policies and the Editorial Policy Checklist.

Statistics

For all statistical analyses, confirm that the following items are present in the figure legend, table legend, main text, or Methods section.

n/a	Confirmed	
☐	☒	The exact sample size (n) for each experimental group/condition, given as a discrete number and unit of measurement
☐	☒	A statement on whether measurements were taken from distinct samples or whether the same sample was measured repeatedly
☐	☒	The statistical test(s) used AND whether they are one- or two-sided<br>*Only common tests should be described solely by name; describe more complex techniques in the Methods section.*
☒	☐	A description of all covariates tested
☐	☒	A description of any assumptions or corrections, such as tests of normality and adjustment for multiple comparisons
☐	☒	A full description of the statistical parameters including central tendency (e.g. means) or other basic estimates (e.g. regression coefficient) AND variation (e.g. standard deviation) or associated estimates of uncertainty (e.g. confidence intervals)
☐	☒	For null hypothesis testing, the test statistic (e.g. F, t, r) with confidence intervals, effect sizes, degrees of freedom and P value noted<br>*Give P values as exact values whenever suitable.*
☒	☐	For Bayesian analysis, information on the choice of priors and Markov chain Monte Carlo settings
☒	☐	For hierarchical and complex designs, identification of the appropriate level for tests and full reporting of outcomes
☒	☐	Estimates of effect sizes (e.g. Cohen's d, Pearson's r), indicating how they were calculated

Our web collection on statistics for biologists contains articles on many of the points above.

Software and code

Policy information about availability of computer code

Data collection	Confocal image were acquired using a Zeiss LSM 700 confocal microscope or Zeiss Axioplan 2.<br>RNA-seq data were obtained with NEBNext Ultra II Directional RNA Library Prep Kit for Illumina, NEBNext Poly(A) mRNA Magnetic Isolation Module, and Nextseq500.<br>Metabolomic data were obtained by using ultra-performance liquid chromatography-tandem mass spectrometry (LCMS-8060, Shimadzu).
Data analysis	All images were analysed and processed using Fiji ver 2.0.0.<br>For RNA-seq, we obtained on average of 30 million reads per biological replicate. We used FASTQC to evaluate the quality of raw single-end reads and trimmed 1 base pair from 3´ end, adaptors and reads of less than 20q base pairs in length from the raw reads using Trim galore 0.6.4 (Babraham Bioinformatics). Reads were aligned with HISAT2 2.1.0 to the BDGP D. melanogaster genome (dm6). Next, Samtools 1.9 and Stringtie 2.0.6 were used to sort, merge, and count reads. The number of trimmed mean of M values (TMM)-normalised fragments per kilobase of combined exon length per one million of total mapped reads (TMM-normalised FPKM value) was calculated with R 3.6.1, Ballgown 2.18.0 and edgeR 3.28.0, and used to estimate gene expression levels.<br>Metabolomic data were analysed by Primary metabolites package ver.2 (Shimadzu).<br>All statistical analyses were carried out using the "R" 3.6.3 software environment.

For manuscripts utilizing custom algorithms or software that are central to the research but not yet described in published literature, software must be made available to editors and reviewers. We strongly encourage code deposition in a community repository (e.g. GitHub). See the Nature Research guidelines for submitting code & software for further information.

April 2020

Data

Policy information about availability of data

All manuscripts must include a data availability statement. This statement should provide the following information, where applicable:

- Accession codes, unique identifiers, or web links for publicly available datasets
- A list of figures that have associated raw data
- A description of any restrictions on data availability

Raw RNA-seq data have been deposited in DNA Data Bank of Japan Sequence Read Archive (Accession number DRA010538; https://www.ncbi.nlm.nih.gov/sra/?term=DRA010538). A part of the raw RNA-seq data is represented in Extend Data Tables 1, 2, and 5. Raw metabolomic data are represented in Extend Data Tables 3 and 4. Other raw data are available from Source Data files.

Field-specific reporting

Please select the one below that is the best fit for your research. If you are not sure, read the appropriate sections before making your selection.

☒ Life sciences ☐ Behavioural & social sciences ☐ Ecological, evolutionary & environmental sciences

For a reference copy of the document with all sections, see nature.com/documents/nr-reporting-summary-flat.pdf

Life sciences study design

All studies must disclose on these points even when the disclosure is negative.

Sample size	We used no statistical methods to predetermine sample size. Sample numbers are described in each figure for each genotype. We chose p-value less than 0.05 and a power level of around 80%. For starvation analysis, we measured at least 55 animals in each group because the over 55 sample number shows power level when p-value less than 0.05, and hazard ratio around 0.5.<br>In metabolic analysis (TAGs, glycaemic levels, feeding amounts), we set sample numbers at least 4, each of which includes 4-30 animals.<br>In qPCR analysis, we used at least 3 samples depending on the standard deviation (SD) to ensure power level.<br>In immunohistochemistry analysis and glucose sensor imaging, we used at least 10 samples.<br>Moreover, these sample sizes have been successfully used in the statistics of previous publications (Ameku, T. et al. 2018; Song, W. et al. 2017; Scopelliti, A. et al. 2019; Miyamoto, T. & Amrein, H. 2019).
Data exclusions	No data were excluded.
Replication	All experiments were performed independently at least twice. Further replicates were included if necessary, for example to account for variability resulting from food batch variation. All attempt at replication were successful, except for RNA-seq data shown in Supplementary Figure 4 because replicate No. 1 of TKg>LacZRNAi exhibited deviation in the expression pattern. We clarify this point in the main text.
Randomization	No specific mathematical algorithm of randomization was used in this study. For all the behavioral and physiological tests, flies of each genotype were collected randomly from the fly bottles where we breed the flies.
Blinding	The investigators were not blinded during data collection as the biological groups were well defined and handled in parallel. Instead of the blinding, we relied upon replication, independent re-analyses/quantifications, and replication by independent technique and genotypes to support our hypothesis.

Reporting for specific materials, systems and methods

We require information from authors about some types of materials, experimental systems and methods used in many studies. Here, indicate whether each material, system or method listed is relevant to your study. If you are not sure if a list item applies to your research, read the appropriate section before selecting a response.

Materials & experimental systems

n/a	Involved in the study	
☐	☒	Antibodies
☐	☒	Eukaryotic cell lines
☒	☐	Palaeontology and archaeology
☐	☒	Animals and other organisms
☒	☐	Human research participants
☒	☐	Clinical data
☒	☐	Dual use research of concern

Methods

n/a	Involved in the study	
☒	☐	ChIP-seq
☒	☐	Flow cytometry
☒	☐	MRI-based neuroimaging

Antibodies

Antibodies used	Primary antibodies<br>Chicken anti-GFP (Abcam, #ab13970, 1:4000 dilution), rabbit anti-RFP (Medical and Biological Laboratories, #PM005, 1:2000 dilution), mouse anti-Prospero (Developmental Studies Hybridoma Bank [DSHB], 1:50 dilution), guinea pig anti-NPF (this study, 1:2000 dilution), rabbit anti-Tk (Veenstra et al., 2008, Song et al. 2014, 1:2000 dilution) , rabbit anti-Bursα (Peabody, et al, 2008, 1:200 dilution) , rabbit anti-sNPF (Lee et al, 2004, 1:1000 dilution) , rabbit anti-AKH (Lee and Park, 2004, 1:1000) , rabbit anti-FOXO (Bai et al, 2013, 1:200 dilution) , guinea pig anti-DILP2 (Okamoto et al, 2015, 1:2000 dilution) , rabbit anti-DILP3 (Veenstra et al, 2008, 1:2000 dilution) , and rabbit anti-DILP5 (Söderberg et al, 2011, 1:1000 dilution), anti-FLAG (Sigma-Aldrich, F1804), anti-HA antibody conjugated with peroxidase (Roche, 12013819001), rabbit anti-pAkt antibody (Cell Signaling Technology, 4060S, 1:1,000 dilution), rabbit anti-AKT antibody (Cell Signaling Technology 9272S, 1:1,000 dilution), mouse anti-β-actin antibody (Santa Cruz Biotechnology, B2008, 1:1000 dilution)<br><br>Secondary antibodies<br>Goat anti-chicken IgG Alexa fluor® 488 (Thermo Fisher Scientific #A11039), Goat anti-rabbit IgG Alexa fluor® 488 (Thermo Fisher Scientific #A11008), Goat anti-mouse IgG Alexa fluor® 488 (Thermo Fisher Scientific #A11001), Goat anti-guinea pig IgG Alexa fluor® 488 (Thermo Fisher Scientific #A11073), Goat anti-rabbit IgG Alexa fluor® 555 (Thermo Fisher Scientific #A11081), Goat anti-mouse IgG Alexa fluor® 546 (Thermo Fisher Scientific #A11003), Goat anti-guinea pig IgG Alexa fluor® 555 (Thermo Fisher Scientific #A21435), Goat anti-guinea pig IgG Alexa fluor® 633 (Thermo Fisher Scientific #A21105), HRP-conjugated secondary antibodies (GE Healthcare, NA934, NA931)
Validation	Anti-NPF antibody was validated in vivo in Drosophila adults using either NPF knockdown or NPF knockout animals. For antibodies widely used by the scientific community, validation has either been performed by the manufacturer or previously reported as cited in the manuscript.

Eukaryotic cell lines

Policy information about cell lines

Cell line source(s)	Drosophila S2 cell (Drosophila Genomics Resource Center, cell line stock #6)
Authentication	While the cell line was not authenticated, Drosophila S2 cell is the only cell line in R.N.'s laboratory and has been maintained over 5 years.
Mycoplasma contamination	The cell line was not tested for mycoplasma contamination.
Commonly misidentified lines (See ICLAC register)	No commonly misidentified cell lines were used in the study.

Animals and other organisms

Policy information about studies involving animals; ARRIVE guidelines recommended for reporting animal research

Laboratory animals	Established Drosophila melanogatser stocks were used.<br>6 days old virgin female flies were used for all fly experiments.<br>The following transgenic and mutant stocks were used: NPFsk1 and NPFRsk8, NPFDf(3R)ED10642 (Kyoto stock center [DGRC] #150266), NPFRDf(3R)BSC464 (Bloomington stock center [BDSC] #24968), AkhKO (a gift from Yi Rao, Peking University School of Life Sciences, China), AkhA, AkhR1 (gifts from Ronald P. Kühnlein, Max-Planck-Institut für Biophysikalische Chemie, Germany), sut1KO (this study), tub>FRT>GAL80>FRT (BDSC# 38879), Otd-FLP (a gift from Daisuke Yamamoto, National Institute of Information and Communications Technology, Japan), Tk-gut-GAL4, UAS-LacZRNAi (gifts from Masayuki Miura, the University of Tokyo, Japan), nSyb-GAL4 (BDSC#51941), Akh-GAL4 (BDSC#25683), dilp2-GAL4 (BDSC#37516), how-GAL4 (BDSC#1767), tj-GAL4 (Kyoto stock center #104055), NPFRKI-T2A-GAL4, NPFRKI-RA/RC-GAL4(BDSC#84672), fbp-GAL4(a gift from Chika Miyamoto and Hubert Amrein, Texas A&M University, USA), sut1KI-T2A-GAL4 (this study), tub-GAL80ts (BDSC#7019), UAS-NPF (a gift from Ping Shen, University of Georgia, USA), UAS-NPFR (a gift from Ping Shen, University of Georgia, USA), UAS-mCD8::GFP (BDSC#32186), UAS-FLII12Pglu-700µδ6 (a gift from Chika Miyamoto and Hubert Amrein), UAS-trans-Tango (BDSC#77480), UAS-sut1::mVenus (this study), CaLexA (BDSC#66542), and tGPH (BDSC#8164).<br>RNAi constructs targeting UAS-NPFRNAiKK (VDRC#108772), UAS-NPFRNAiTRiP (BDSC#27237), UAS-NPFRRNAiKK (VDRC#107663), UAS-NPFRRNAiTRiP (BDSC#25939), UAS-sut1RNAi (VDRC#104983), UAS-sut1RNAiTRiP (BDSC#65964), UAS-Glut1RNAi (VDRC#101365), UAS-AkhRNAi (VDRC#105063), UAS-AkhRRNAi (VDRC#109300), UAS-BmmRNAi (VDRC#37877), UAS-dHSLRNAi (VDRC#109336), UAS-dilp2RNAi (VDRC#102158), UAS-dilp3RNAi (VDRC# 106512), UAS-dilp5RNAi (VDRC# 105004), and UAS-dskRNAi (VDRC# 106592).
Wild animals	Wild animals were not used in this study.
Field-collected samples	Field-collected samples were not used in this study.
Ethics oversight	No ethical approval is required for D. melanogaster study in Japan. The usage of genetically modified Drosophila melanogaster was approved by the Committee on the usage of genetically modified organisms in University of Tsukuba, The University of Tokyo, Kumamoto University, Tohoku University, and National Institute of Genetics.

Note that full information on the approval of the study protocol must also be provided in the manuscript.
